# A nitrogenase-like enzyme is involved in the novel anaerobic assimilation pathway of a sulfonate, isethionate, in the photosynthetic bacterium *Rhodobacter capsulatus*

**DOI:** 10.1128/msphere.00498-24

**Published:** 2024-08-27

**Authors:** Yoshiki Morimoto, Kazuma Uesaka, Yuichi Fujita, Haruki Yamamoto

**Affiliations:** 1 Graduate School of Bioagricultural Sciences, Nagoya University, Nagoya, Japan; University of Michigan, Ann Arbor, Michigan, USA

**Keywords:** nitrogenase, sulfonates, photosynthetic bacteria, anaerobic catabolic pathways

## Abstract

Prokaryotes contribute to the global sulfur cycle by using diverse sulfur compounds as sulfur sources or electron acceptors. In this study, we report that a nitrogenase-like enzyme (NFL) and a radical SAM enzyme (RSE) are involved in the novel anaerobic assimilation pathway of a sulfonate, isethionate, in the photosynthetic bacterium *Rhodobacter capsulatus*. The *nflHDK* genes for NFL are localized at a locus containing genes for known sulfonate metabolism in the genome. A gene *nflB* encoding an RSE is present just upstream of *nflH*, forming a small gene cluster *nflBHDK*. Mutants lacking any *nflBHDK* genes are incapable of growing with isethionate as the sole sulfur source under anaerobic photosynthetic conditions, indicating that all four NflBHDK proteins are essential for the isethionate assimilation pathway. Heterologous expression of the *islAB* genes encoding a known isethionate lyase that degrades isethionate to sulfite and acetaldehyde restored the isethionate-dependent growth of a mutant lacking *nflDK*, indicating that the enzyme encoding *nflBHDK* is involved in an isethionate assimilation reaction to release sulfite. Furthermore, the heterologous expression of *nflBHDK* and *ssuCAB* encoding an isethionate transporter in the closely related species *R. sphaeroides*, which does not have *nflBHDK* and cannot grow with isethionate as the sole sulfur source, conferred isethionate-dependent growth ability to this species. We propose to rename *nflBHDK* as *isrBHDK* (isethionate reductase). The *isrBHDK* genes are widely distributed among various prokaryote phyla. Discovery of the isethionate assimilation pathway by IsrBHDK provides a missing piece for the anaerobic sulfur cycle and for understanding the evolution of ancient sulfur metabolism.

**IMPORTANCE:**

Nitrogenase is an important enzyme found in prokaryotes that reduces atmospheric nitrogen to ammonia and plays a fundamental role in the global nitrogen cycle. It has been noted that nitrogenase-like enzymes (NFLs), which share an evolutionary origin with nitrogenase, have evolved to catalyze diverse reactions such as chlorophyll biosynthesis (photosynthesis), coenzyme F_430_ biosynthesis (methanogenesis), and methionine biosynthesis. In this study, we discovered that an NFL with unknown function in the photosynthetic bacterium *Rhodobacter capsulatus* is a novel isethionate reductase (Isr), which catalyzes the assimilatory degradation of isethionate, a sulfonate, releasing sulfite used as the sulfur source under anaerobic conditions. Isr is widely distributed among various bacterial phyla, including intestinal bacteria, and is presumed to play an important role in sulfur metabolism in anaerobic environments such as animal guts and microbial mats. This finding provides a clue for understanding ancient metabolism that evolved under anaerobic environments at the dawn of life.

## INTRODUCTION

Nitrogenase is a metalloenzyme responsible for biological nitrogen fixation, catalyzing the reduction of atmospheric nitrogen molecules (N_2_) to ammonia (NH_3_), which provides a nitrogen source to support most organisms and plays a critical role in the global nitrogen cycle (1). Nitrogenase comprises three subunits (NifH, NifD, and NifK) (2, 3). NifH functions as the reductase component as a homodimer (Fe protein), and NifD and NifK form a heterotetramer (MoFe protein) to function as the catalytic component.

Nitrogenase-like enzymes (*nif*-like enzymes; NFLs) are a group of metalloenzymes that share high structural similarity with nitrogenase and catalyze a variety of reductions different from the conversion of N_2_ to NH_3_ (4–6). Dark-operative protochlorophyllide oxidoreductase (DPOR; BchLNB/ChlLNB) has been identified as a first NFL (7–9), followed by the identification of a second NFL, chlorophyllide *a* oxidoreductase (COR; BchXYZ) (10). These NFLs are involved in (bacterio)chlorophyll biosynthesis.

Genes for NFLs are distributed among various prokaryotes. Raymond *et al*. (4) classified NFLs into five groups (I–V) on the basis of molecular phylogenetic analysis (4). Three types (Mo, V, and Fe) of nitrogenases were classified into Groups I, II, and III. DPOR and COR formed Group V (including chloroplast DPOR). The remaining Group IV consisted of only NFLs of unknown functions at the time this phylogenetic classification was proposed. Recently, two NFLs of Group IV were identified as biosynthetic enzymes for the cofactor F_430_ (Ni^2+^-sirohydrochlorin *a*,*c*-diamide reductive cyclase, CfbCD) and methionine (methylthioalkane reductase, MarHDK), revealing the functional diversity of Group IV NFLs (11–13).

The NFLs consist of three subunits homologous to NifH, NifD, and NifK (only Cfb lacks a NifK homolog). The NifH homologs (NifH, BchL/ChlL, BchX, CfbC, and MarH) function as the reductase components, and the NifD and NifK homologs (NifEN, BchNB/ChlNB, CfbD, and MarDK) form a heterotetramer (only CfbD is a homodimer) as the catalytic components to catalyze the reduction of their individual substrates using electrons from the NifH homologs. The reductase component, the NifH homolog, carries a [4Fe-4S] cluster that serves as the redox center for electron transfer to the cognate catalytic component (3, 12, 14–17). The catalytic component carries at least a pair of [4Fe-4S] clusters, and the electrons from the reductase component are used to reduce the substrate via the [4Fe-4S] clusters (3, 9, 12, 18, 19). Thus, nitrogenase and NFLs catalyze various reductions through inter- and intramolecular electron transfers based on a common architecture with their metal clusters as the redox centers. These metal clusters, particularly the [4Fe-4S] cluster of the reductase component, are rapidly and irreversibly destroyed by oxygen (O_2_) (3, 17, 20). Therefore, an anaerobic environment is required for the action of NFLs, similar to nitrogenases.

*Rhodobacter capsulatus* is a purple non-sulfur photosynthetic bacterium that performs anoxygenic photosynthesis under anaerobic conditions. *R. capsulatus* has the two NFLs, DPOR and COR, in the late steps of bacteriochlorophyll biosynthesis in addition to two types of nitrogenases (Mo- and Fe-types) (4). In the genome of *R. capsulatus,* three genes for a third NFL (Group IV) remain with an unknown function. These genes, rcc02236, rcc02235, and rcc02234, show significant similarity to the nitrogenase three subunits NifH, NifD, and NifK, forming an operon similar to *nifHDK*, which are tentatively call *nflH*, *nflD*, and *nflK*, respectively (Fig. 2; Figs. S1 to S3).

**Fig 1:**
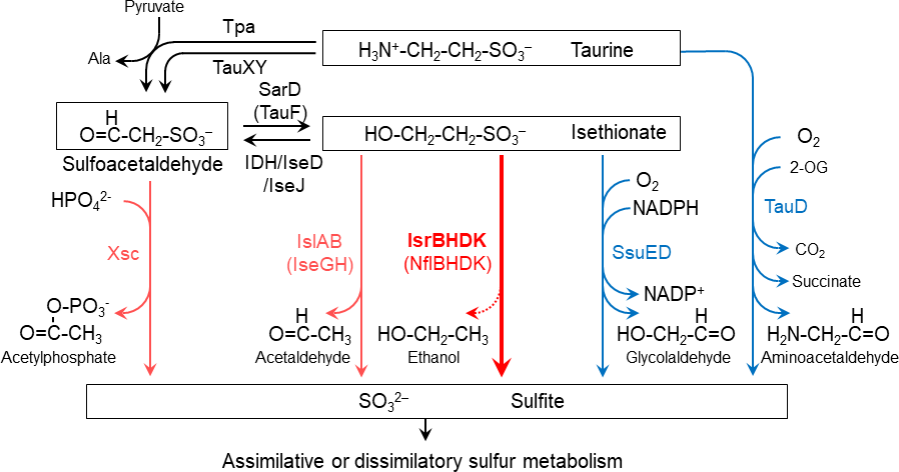
Metabolic pathways of taurine and isethionate in various bacteria. Oxygen-dependent and oxygen-independent desulfurization reactions are shown in blue and red, respectively. The new isethionate metabolic pathway (isethionate reductase; IsrBHDK) identified in this study is shown with a thick red arrow and in bold. Isethionate reductase releases sulfite from isethionate. However, the carbon product ethanol (EtOH) has not yet been experimentally identified (dotted line).

Sulfonates are organic sulfur compounds with alkyl or aryl groups and sulfo (-SO_3_^-^) groups, found in various environments including soil, marine and freshwater, as well as their sediments, microbial mats, and animal intestines (21–23). Among these sulfonates, low molecular weight sulfonates like taurine (2-aminoethanesulfonate) and isethionate (2-hydroxyethanesulfonate) are naturally produced through organic decomposition and microbial metabolism (Fig. 1). For prokaryotes, these sulfonates serve not only as sulfur and carbon sources but also function as terminal electron acceptors in anaerobic respiration (22–24). Out of 100 randomly isolated bacterial strains from freshwater, soil, and freshwater sediments, 94 strains were reported to utilize taurine as a sulfur source, and 96 strains were reported to utilize isethionate as a sulfur source, demonstrating the widespread utilization of sulfonates as sulfur sources in nature (21). Additionally, approximately 600 Tg of dissolved organic sulfur, including taurine and isethionate, is estimated to be released into the ocean each year through biological processes (25). From these instances, it can be assumed that sulfonate-degrading enzymes are not only significant as prokaryotic metabolic enzymes but also play an essential role in the global cycling of carbon and sulfur.

In prokaryotes, four pathways for desulfurization of taurine and isethionate are known so far (Fig. 1) (26–30). The first pathway is catalyzed by sulfoacetaldehyde acetyltransferase (Xsc) (27). Sulfoacetaldehyde is produced by the deamination of taurine (taurine pyruvate aminotransferase; Tpa or TauXY) (31, 32) or the oxidation of isethionate (isethionate dehydrogenase; IDH or IseJ or IseD) (32–34). The second pathway is the conversion of isethionate to sulfite and acetaldehyde by isethionate lyase IslAB, which is an oxygen-independent reaction (28, 29). The third pathway is catalyzed by the NAD(P)H-dependent monooxygenase SsuED, which produces sulfite and glycolaldehyde from isethionate in an oxygen-dependent manner (26). The fourth pathway is the oxygen-dependent conversion of taurine to sulfite by TauD (30). These four metabolic pathways share the common feature of cleaving the C-S bond of sulfonates to produce sulfite. Generated sulfite is thought to be assimilated into cysteine via sulfur assimilation pathways that are common among many prokaryotes. *R. capsulatus* possesses Tpa and Xsc, allowing the production of sulfite from taurine to use the released sulfite as a sulfur source (35). However, even in mutants in which both genes for Tpa and Xsc were disrupted, the ability to metabolize taurine was not completely lost (35). Furthermore, proteins showing significant similarity to IslAB, SsuED, and TauD were not found in the *R. capsulatus* genome, suggesting the existence of a novel, previously unknown sulfonate metabolic pathway in *R. capsulatus*.

In this study, we report that the third NFL in *R. capsulatus* is involved in a novel pathway for the use of isethionate as a sulfur source in anaerobic photosynthetic growth. The possible involvement of the third NFL in sulfonate metabolism was inferred from the known sulfonate metabolic genes existing at a locus in the vicinity of the NFL genes. Analyses of the phenotype of targeted mutants, transcriptome, and genome profiling suggested that the NFL is involved in a novel anaerobic isethionate metabolic pathway. This hypothesis was confirmed by the heterologous expression of the genes for the NFL and a putative isethionate transporter in a closely related species, *R. sphaeroides*, resulting in the ability to use isethionate as a sulfur source in this organism. This metabolic pathway, which is widely distributed among various prokaryotic phyla, is thought to play an important role in sulfur metabolism in anaerobic environments. This discovery not only shows the novel function of the NFL but also provides a new perspective on sulfur metabolism at the dawn of life, which evolved under anaerobic environments.

## RESULTS

### The *nflHDK* genes for the third NFL

**Fig 2:**
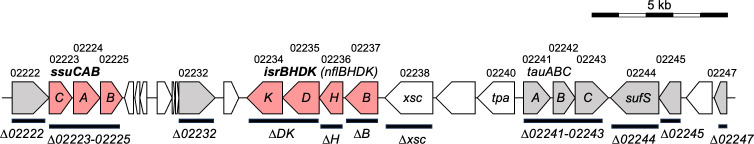
Gene arrangement of the 26-kb locus containing *isrBHDK* of the *R. capsulatus* genome. Genes shown by pink and gray are conserved specifically in the species carrying the *isrBHDK (nflBHDK)* genes in the genome profiling analysis (Table S3) and show high values (>100) of the Ise/Sul ratio (Table 1). Genes shown by pink are the minimal set that conferred isethionate-dependent growth ability to *R. sphaeroides* (Table 2). The thick horizontal bars indicate the regions that were deleted in the targeted mutants (Fig. 3; Table 2). Names of mutants are shown below the thick horizontal bars. The five-digit numbers on the gene map indicate the numbers following “rcc” of the locus tag.

The three genes, rcc02236, rcc02235, and rcc02234, that we focus on in this study encode functionally unknown NFL proteins that show significant similarity to the nitrogenase three subunits NifH, NifD, and NifK (44.1%, 16.8%, and 16.4% from *Azotobacter vinelandii*, respectively), forming an operon *nflHDK* (Fig. 2; Figs. S1 to S3). In addition, a gene (rcc02237) immediately upstream of *nflH* encodes a protein that shows features of a radical SAM enzyme (RSE). We call this gene *nflB* after *nifB* (13.8% similarity to NifB of *A. vinelandii*; Fig. S4), which encodes an RSE involved in FeMo-co biosynthesis in the nitrogenase system. In the genome of *R. capsulatus*, genes for known sulfonate metabolism, such as *xsc* and *tpa*, are encoded in the vicinity of the *nflBHDK* gene cluster, implying that the proteins encoded by *nflBHDK* may also be involved in sulfonate metabolism (Fig. 2) (36).

### A novel alternative *xsc*-independent metabolic pathway for isethionate

**Fig 3:**
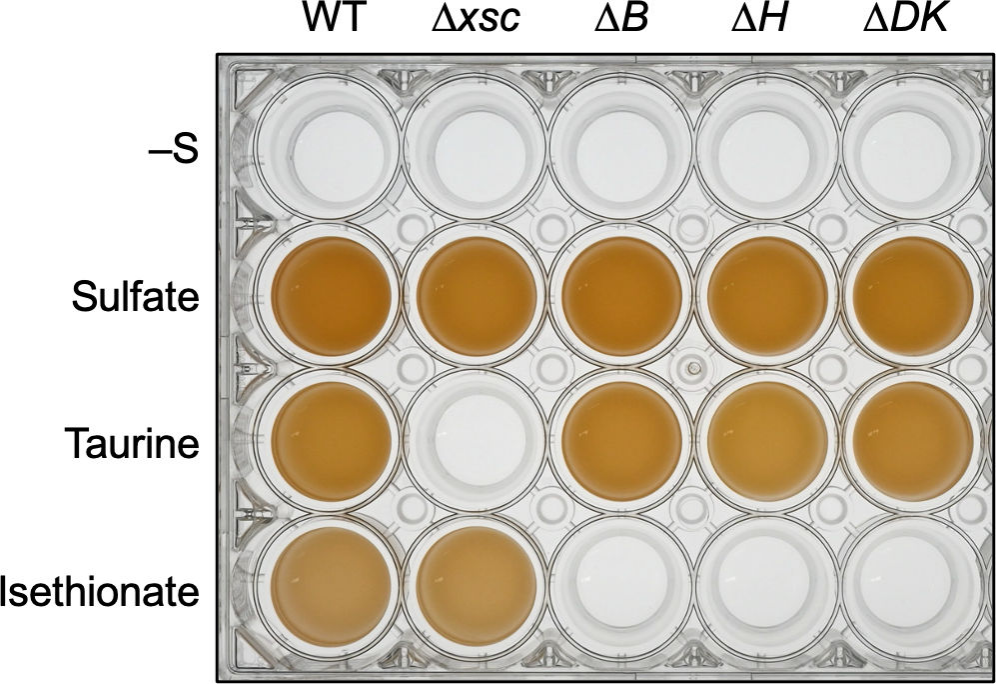
Growth of WT and four targeted mutants under anaerobic photosynthetic conditions with three sulfur compounds. WT and the four mutants were grown with various sulfur sources in screw-capped test tubes under anaerobic photosynthetic conditions. The cultures without sulfur (–S), with sulfate, taurine, and isethionate were incubated for 120 hours, 48 hours, 72 hours, and 120 hours, respectively. The resultant cultures were transferred into a 24-well plate to clearly show their growth.

To elucidate the function of these genes in terms of sulfonate metabolism, we first examined photosynthetic growth of *R. capsulatus* with two sulfonates, taurine and isethionate, as the sole sulfur sources (Fig. 3). *R. capsulatus* WT (see *Materials and Method*s) grew well with both sulfonates and sulfate, indicating that *R. capsulatus* can utilize both taurine and isethionate as the sole sulfur sources. To confirm the metabolic pathway of these sulfonates in *R. capsulatus*, we constructed an *xsc*-knockout mutant, *∆xsc*, and examined photosynthetic growth (Fig. 3). In contrast to the loss of the growth ability of ∆*xsc* in taurine medium, *∆xsc* grew well with isethionate as the sole sulfur source, indicating that Xsc is essential for taurine assimilation, but not for isethionate. Given that *R. capsulatus* does not have genes corresponding to the *islAB* and *ssuED* genes (Fig. 1), this result suggested the presence of a novel alternative *xsc*-independent metabolic pathway for isethionate in *R. capsulatus* under anaerobic photosynthetic conditions.

### Genes induced by isethionate

Next, to obtain clues to genes involved in the novel isethionate metabolic pathway in *R. capsulatus*, transcriptome analysis (RNA-seq) was performed to globally extract genes that are specifically induced under growth conditions to use isethionate as a sulfur source. We assumed that genes involved in isethionate metabolism are transcriptionally repressed when growing with sulfate as the sulfur source and induced when growing with isethionate as the sole sulfur source. *R. capsulatus* WT was cultivated photosynthetically in a medium containing sulfate or isethionate as the sole sulfur source. The ratios of transcript levels grown with isethionate to those grown with sulfate (the Ise/Sul ratio) were estimated for all genes. Thirty genes were extracted with Ise/Sul ratios greater than 100 (Table 1), 23 of which were located at the chromosomal locus spanning approximately 26 kb from rcc02222 to rcc02247 (Fig. 2; Fig. S5). The *nflBHDK* gene cluster contained this chromosomal locus and showed high Ise/Sul ratios (1,300 to 5,000). In addition, other genes in the 26-kb locus, such as the *tauABC* genes for the ABC-type taurine transporter (rcc02241–rcc02243), genes for the ABC-type sulfonate transporter (rcc02223–rcc02225), and *sufS* for cysteine desulfurase (rcc02244), also showed high Ise/Sul ratios. Four genes, rcc02222 (monooxygenase-type enzyme), rcc02228, rcc02229, and rcc02232 (FAD dependent oxidoreductase), whose functions are unknown, also showed Ise/Sul ratios of more than 2,000 (Table 1). In contrast, the Ise/Sul ratios of *xsc* and *tpa* were less than 100, even though these genes are located at just upstream of *nflBHDK* (Fig. S5). The RNA-seq results indicate that *nflBHDK* is involved in a novel isethionate metabolic pathway.

**TABLE 1:** List of genes specifically induced on the medium with isethionate as the sole sulfur source (>100 Ise/Sul ratio) under anaerobic photosynthetic heterotrophic conditions

Locus tag (rcc)^*a*^	Annotation^*b*^	Ise/Sul Ratio^*c*^	FDR^*d*^
02113	Ornithine cyclodeaminase-1	129.0	3.04E-70
02143	Hypothetical protein	4011.0	2.12E-73
02222	ntaA; nitrilotriacetate monooxygenase, component A	4968.0	1.62E-83
02223	ABC transporter, permease protein (SsuC)	4662.3	7.74E-53
02224	ABC transporter, substrate-binding protein (SsuA)	3422.2	6.14E-130
02225	ABC transporter, ATP-binding protein (SsuB)	1259.4	1.26E-25
02226	Conserved hypothetical protein	795.1	3.93E-44
02227	Conserved hypothetical protein	1148.0	6.71E-24
02228	Conserved hypothetical protein	2810.0	2.53E-48
02229	Protein of unknown function DUF1234	2816.7	1.29E-119
02230	Membrane protein, putative	423.8	7.88E-20
02231	Hypothetical protein	407.9	1.16E-19
02232	FAD-dependent oxidoreductase	3987.9	1.66E-117
02233	Conserved hypothetical protein	1386.9	1.19E-52
02234	Oxidoreductase/nitrogenase, component 1 (IsrK)	1748.9	5.70E-64
02235	Oxidoreductase/nitrogenase, component 1 (IsrD)	1148.9	4.24E-88
02236	nifH2; nitrogenase iron protein-2 (IsrH)	1819.7	8.72E-65
02237	Radical SAM family protein (IsrB)	4787.6	3.07E-76
02241	tauA; taurine ABC transporter, periplasmic taurine-binding protein TauA	931.6	1.56E-48
02242	tauB; taurine ABC transporter, ATP-binding protein TauB	488.4	2.52E-30
02243	tauC; taurine ABC transporter, permease protein TauC	687.1	5.85E-26
02244	sufS2; cysteine desulfurase-2	1421.7	6.32E-110
02245	major membrane protein I	1292.6	1.06E-149
02246	serine O-acetyltransferase-2	1215.4	1.78E-84
02247	Rhodanese domain protein	1005.3	5.27E-37
02281	Dimethyl sulfoxide reductase, A subunit	264.3	2.20E-16
02535	Sulfate ABC transporter, permease protein CysW	182.8	3.24E-43
02536	Sulfate ABC transporter, permease protein CysT	289.4	6.28E-46
02647	Bifunctional protein PutA	116.1	6.42E-168
02744	Sulfate/thiosulfate ABC transporter, periplasmic sulfate/thiosulfate-binding protein	4603.8	4.86E-260

^*a*^ The five-digit number indicates the number following the locus tag of rcc.

^*b*^ Annotations in the GenBank data base. The names of proteins proposed in this work are shown in parentheses.

^*c*^ The ratio of transcript levels of the gene in cells grown in media containing isethionate (Ise) and sulfate (Sul) as the sole sulfur sources.

^*d*^ FDR calculated by DEseq2.

### Targeted mutants for *nflBHDK*

Then, targeted mutants for *nflB*, *nflH*, and *nflDK* (*∆B*, *∆H*, and *∆DK*, respectively) were constructed (Fig. 2), and photosynthetic growth was examined in the isethionate medium (Fig. 3). All three mutants lost the ability to grow in the isethionate medium. These results indicated that all four *nflBHDK* genes are essential for growth using isethionate as the sole sulfur source.

**Fig 4:**
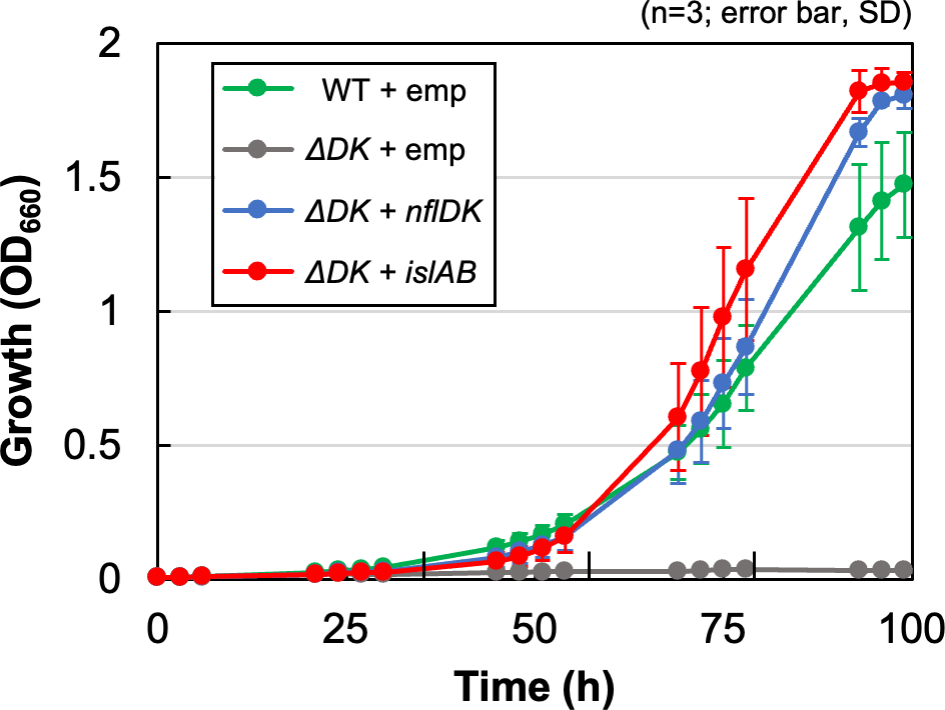
Complementation of the growth ability of *∆DK* in the isethionate medium with expression of *islAB*. Transconjugants carrying the empty vector (WT/∆*DK* +emp) and *∆DK* carrying *nflDK* (∆*DK +nflDK*) and *islAB* (∆*DK +islAB*) were grown in isethionate media under anaerobic photosynthetic conditions. Growth was monitored with optical density at 660 nm (OD_660_).

To specify the reaction involving NflBHDK, an expression plasmid of the *islAB* genes encoding isethionate lyase, which catalyzes the degradation of isethionate to sulfite and acetaldehyde from the enterobacterium *Bilophila wadsworthia* 3.1.6, was introduced into the *∆DK* mutant. The transconjugant, *∆DK +islAB*, carrying the expression plasmid, recovered growth in the isethionate medium (Fig. 4). This result indicates that the isethionate lyase reaction can substitute for a reaction involving NflDK to support isethionate-dependent growth. Given that sulfite is converted to sulfide by sulfite reductase in *R. capsulatus* to use as a sulfur source, this result indicates that the reaction catalyzed by NflDK, which can be replaced by IslAB, contains at least the release of sulfite from isethionate. Furthermore, considering that NFLs catalyze a variety of reductions, the *nflHDK* genes are most likely to encode subunits of a novel enzyme, isethionate reductase, that catalyzes the reductive cleavage of the C-S bond of isethionate. On the basis of these results, we propose to rename *nflBHDK* as *isrBHDK* after isethionate reductase.

### Genome profiling analysis

To clarify whether the four genes *isrBHDK* are sufficient for the availability of isethionate as a sulfur source or other genes are required, genome profiling analysis was performed. In this *in silico* analysis, genes specifically conserved in organisms carrying *isrBHDK* are extracted by genome comparison among closely related species. Eight species, including *R. capsulatus* SB1003 (*R. capsulatus*_B in the GTDB classification), were selected as a group in which *isrBHDK* genes are conserved. Five species, including *R. capsulatus* DSM1710, and 17 species, including *R. sphaeroides* 2.4.1 (*R. capsulatus* and *Celeibacter*_A *sphaeroides* in the GTDB classification, respectively) were selected as the other group in which *isrBHDK* genes are missing (Fig. S7; Tables S1 and S2). Using the genome information of these 30 species, 121 genes were extracted as conserved only in the eight species carrying *isrBHDK* genes (Table S3). Interestingly, 11 of the 121 genes were located in the 26-kb gene cluster containing *isrBHDK*, with Ise/Sul ratios of more than 100 in *R. capsulatus* (Fig. 2). We then regarded the proteins encoded by the 11 genes as strong candidates acting together with IsrBHDK, and targeted mutants were constructed for all 11 genes. For the two sets of genes for the ABC-type transporter in the 11 genes, a single targeted mutant with all three genes removed was constructed rather than three targeted mutants lacking the individual genes. Then, the seven targeted mutants were constructed and examined for photosynthetic growth in the isethionate medium (Fig. 2; Fig. S6). Only one mutant ∆rcc02223-rcc02225 lacking the three genes (rcc02223, rcc02224, and rcc02225) encoding subunits of the ABC-type sulfonate transporter failed to grow in the isethionate medium other than the three mutants for *isrBHDK* (Fig. S6; Table 2). This result indicates that the three genes for the putative isethionate transporter are essential for growth in the isethionate medium and that the transporter acts with IsrBHDK to support photosynthetic growth using isethionate as the sole sulfur source. Because the three subunits of the ABC-type sulfonate transporter (rcc02223, rcc02224, and rcc02225) show high similarity to those of subunits of the ABC-type sulfonate transporter SsuC, SsuA, and SsuB from *E. coli* with a sequence similarity of 39.2%, 28.5%, and 45.7%, respectively, we propose to designate these three genes, rcc02223, rcc02224, and rcc02225, as *ssuC*, *ssuA*, and *ssuB*, respectively, in *R. capsulatus* SB1003 (37).

**TABLE 2:** List of genes conserved specifically in *R. capsulatus* identified by genome profiling analysis and specifically induced on the isethionate medium as the sole sulfur source (>100 Ise/Sul ratio) and the growth ability of the targeted disrupted mutants under anaerobic photosynthetic heterotrophic conditions on the isethionate medium

Locus tag (rcc)^*a*^	Annotation^*b*^	Ise/Sul ratio^*c*^	Growth on Ise culture^*d*^
02222	ntaA; nitrilotriacetate monooxygenase, component A	4968.0	**+**
02223	ABC transporter, permease protein (SsuC)	4662.3	–
02224	ABC transporter, substrate-binding protein (SsuA)	3422.2	
02225	ABC transporter, ATP-binding protein (SsuB)	1259.4	
02232	FAD-dependent oxidoreductase	3987.9	**+**
02234	Oxidoreductase/nitrogenase, component 1 (IsrK)	1748.9	–
02235	Oxidoreductase/nitrogenase, component 1 (IsrD)	1148.9	
02236	nifH2; nitrogenase iron protein-2 (IsrH)	1819.7	–
02237	Radical SAM family protein (IsrB)	4787.6	–
02241	tauA; taurine ABC transporter, periplasmic taurine-binding protein TauA	931.6	**+**
02242	tauB; taurine ABC transporter, ATP-binding protein TauB	488.4	
02243	tauC; taurine ABC transporter, permease protein TauC	687.1	
02244	sufS2; cysteine desulfurase-2	1421.7	**+**
02245	Major membrane protein I	1292.6	**+**
02247	Rhodanese domain protein	1005.3	**+**

^*a*^ The five-digit number indicates the number following the locus tag of rcc.

^*b*^ Annotations in the GenBank database. The names of proteins proposed in this work are shown in parentheses.

^*c*^ The ratio of transcript levels of the gene in cells grown in media containing isethionate (Ise) and sulfate (Sul) as the sole sulfur sources.

^*d*^ Growth ability of the mutant lacking the gene(s) in the isethionate medium under anaerobic photosynthetic conditions.

### Heterologous expression of *isrBHDK* in *R. sphaeroides*

**Fig 5:**
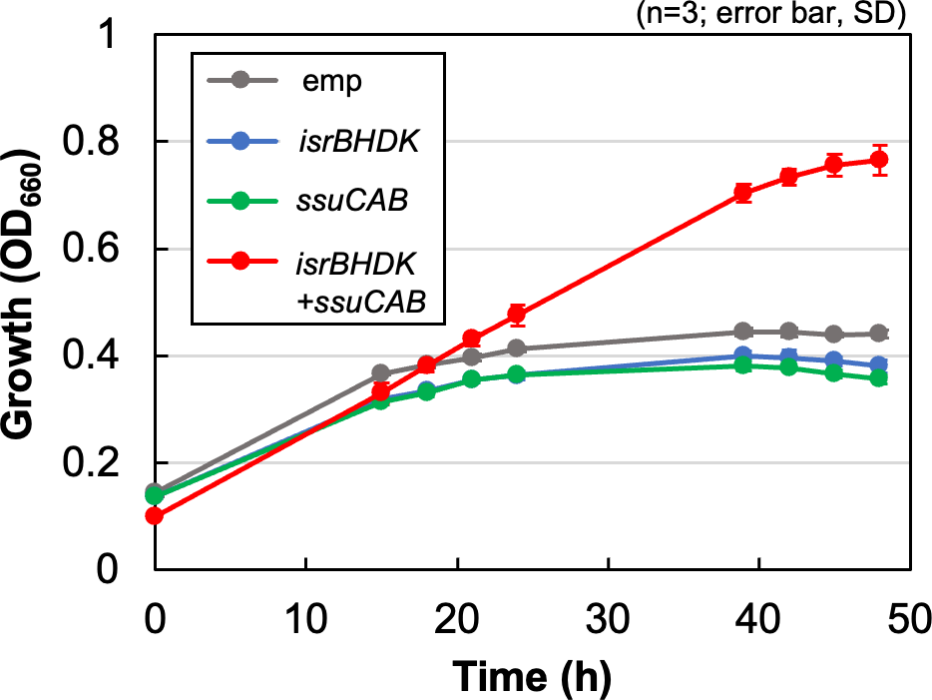
Expression of *isrBHDK* and *ssuCAB* conferred the isethionate assimilation ability to *R. sphaeroides*. Plasmids expressing *isrBHDK*, *ssuCAB* (rcc02223-02225), and both gene clusters of *isrBHDK* and *ssuCAB* (*isrBHDK +ssuCAB)* were introduced into *R. sphaeroides* by conjugation, and growth of the transconjugants was monitored by OD_660_ in the culture medium containing isethionate as the sole sulfur source under anaerobic photosynthetic conditions. The transconjugant carrying only the empty vector (emp) was the negative control.

Genome profiling and reverse genetic analysis suggest that IsrBHDK and SsuCAB are sufficient to use isethionate as the sole sulfur source. Next, we examined whether heterologous expression of these seven genes confers the ability to use isethionate as the sole sulfur source to a different host organism. *R. sphaeroides* 2.4.1, which is a close relative of *R. capsulatus* but lacks the *isrBHDK* genes, is unable to grow in media containing isethionate as the sole sulfur source (Fig. S8). A shuttle vector expressing *isrBHDK* and *ssuCAB* of *R. capsulatus* was introduced into *R. sphaeroides*, and the transconjugant IsrBHDK +SsuCAB was isolated. IsrBHDK +SsuCAB showed significantly enhanced growth in the isethionate medium compared with the control transconjugant carrying the empty vector (Fig. 5). Furthermore, such growth enhancement was not observed in the transconjugant carrying a plasmid expressing only either *isrBHDK* or *ssuCAB*. The results indicate that isethionate in the medium is taken up by the SsuCAB transporter, IsrBHDK releases sulfite from isethionate, and sulfite is used as the sulfur source. It was also strongly suggested that the four genes *isrBHDK* are the minimum gene set required for the novel isethionate assimilatory enzyme Isr to function.

### Complex formation of IsrD and IsrK

**Fig 6:**
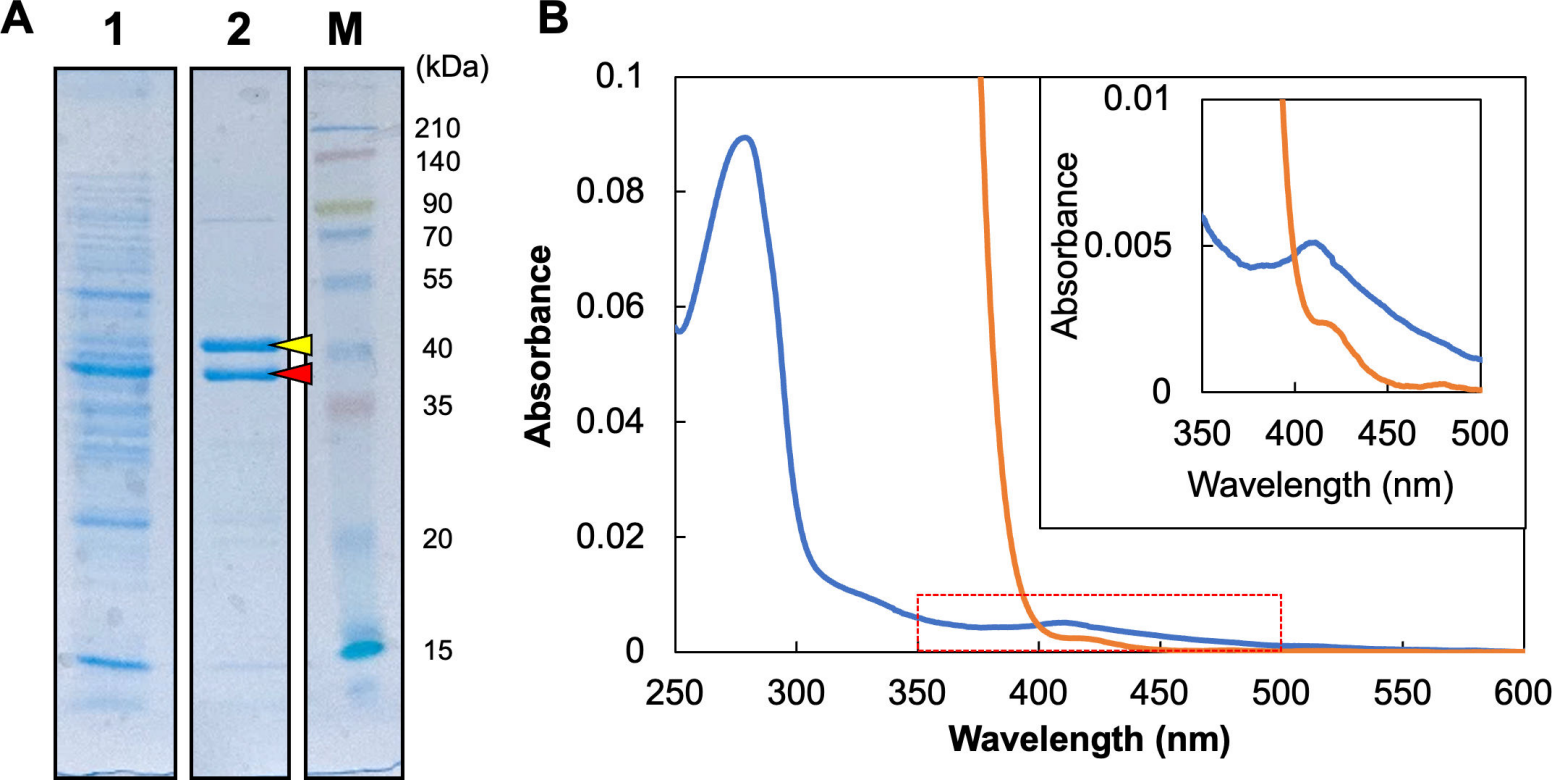
Purification of 6xHis-IsrD and the absorption spectra. (**A**) 6xHis-IsrD protein extracted from the *R. capsulatus* transconjugant carrying the expression plasmid for 6xHis-IsrD was purified using an affinity column and confirmed by SDS-PAGE. Each lane shows the supernatant fraction (lane 1), the purified fraction (lane 2), and molecular weight marker (lane M; 3-color Prestained XL-Ladder. APRO science, Japan). Each lane was loaded with 5 µg of the protein. Two bands (red and yellow arrows) were excised and subjected to mass spectrometry. (**B**) The absorption spectra of the purified 6xHis-IsrDK protein. The isolated and reduced (by dithionite) forms (0.35 mg protein/mL) are shown as blue and orange traces, respectively. The inset is the enlarged panel of the wavelength region from 350 nm to 500 nm.

To elucidate the biochemical properties of Isr, as a first attempt, IsrD and IsrK were overexpressed in *R. capsulatus*. In this expression system, IsrD was expressed with an affinity tag (6xHis) attached to the N-terminus for purification. When the 6xHis-IsrD was purified with an affinity column from the crude supernatant fraction, a protein with an apparent molecular mass of 44 kDa was co-purified (Fig. 6A), and mass spectroscopy identified that the 44-kDa protein was IsrK. This result indicated clearly that IsrD and IsrK form a stable complex. The absorption spectrum of the purified IsrDK complex showed a broad peak around 410 nm, which is characteristic of iron–sulfur proteins. The absorption peak of the as-isolated IsrDK decreased when dithionite was added (Fig. 6B). These results indicated that the IsrDK complex has redox-active iron–sulfur clusters, similar to the nitrogenase MoFe protein and the NB protein of DPOR.

## DISCUSSIONS

**Fig 7:**
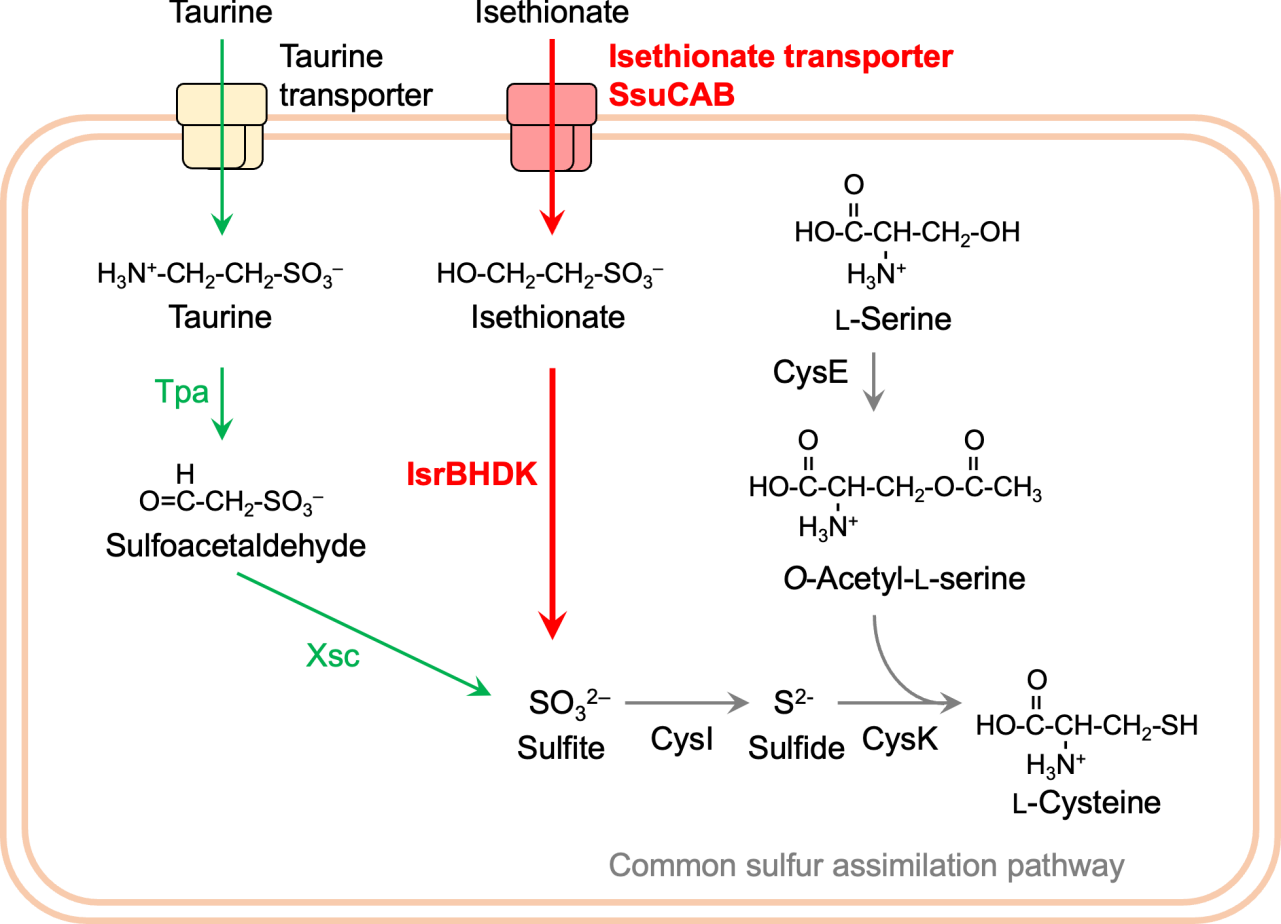
Metabolic pathway of isethionate and taurine in *R. capsulatus* SB1003. Metabolic pathways specific for isethionate and taurine are shown in red and green, respectively. Isethionate reductase (IsrBHDK) and isethionate transporter (SsuCAB) identified in this study are shown in bold. SsuCAB uptakes isethionate in the medium to the cytoplasm, and isethionate reductase degrades isethionate to release sulfite, followed by the sulfur assimilation pathways common to many bacteria indicated by gray arrows.

In this study, we demonstrate that the NFL (IsrHDK) and the RSE (IsrB) in *R. capsulatus* potentially function as the novel enzyme isethionate reductase Isr under anaerobic conditions. IsrHDK is the third NFL whose function was identified after the second NFL, COR, in *R. capsulatus*. The IsrB protein is essential for this enzyme activity, suggesting that IsrHDK functions together with IsrB. In addition, we identified the ABC-type isethionate transporter SsuCAB in *R. capsulatus*. The cooperative actions of IsrBHDK and SsuCAB allow *R. capsulatus* to use isethionate in the environment as a sulfur source (Fig. 7).

### Cysteine conservation in the amino acid sequences of IsrHDK

Based on the structural features of nitrogenases and NFLs, DPOR and COR, which have been biochemically studied, Isr may consist of two components: a reductase component (a IsrH dimer) and a catalytic component (a IsrDK heterotetramer) (7–10, 17, 19). The co-purification of IsrK with IsrD during its purification indicates that IsrDK forms a heteromeric complex similar to nitrogenases, DPOR/COR, and other NFLs (Fig. 6). The conservation of Cys residues, which serve as ligands for iron–sulfur clusters in various iron–sulfur proteins, provides an important clue for predicting the structure and function of metal clusters retained in NFLs. The NifH homolog IsrH has two fully conserved Cys residues (Cys99 and Cys135) that hold a [4Fe-4S] cluster in the nitrogenase Fe protein (NifH), in addition to the full conservation of the ATP-binding motif (Fig. S1) (3). Thus, IsrH, like other NifH homologs, is expected to hold a single [4Fe-4S] cluster in a homodimer and functions as a reductase component that transfers electrons to the catalytic component IsrDK, which is coupled with ATP hydrolysis (3, 16, 38).

Furthermore, absorption spectra of the purified IsrDK complex indicated the presence of iron–sulfur clusters in IsrDK complex (Fig. 6). IsrD, the NifD homolog, has only a single conserved Cys74 corresponding to Cys88 of the three Cys residues (Cys62, Cys88, and Cys154) in NifD of *A. vinelandii* involved in P-cluster chelation in the MoFe protein (Fig. S2) (3). The other two residues corresponding to Cys62 and Cys154 are conserved as Arg and Pro/Ser in IsrD, respectively. The single Cys conservation is in contrast to that of all other NifD homologs (CfbD, MarD, NfaD (39), BchN/ChlN, and BchY) has three conserved Cys in the corresponding region. Regarding the NifK homolog and IsrK, the three Cys (Cys70, Cys95, and Cys153 in NifK of *A. vinelandii*), which are involved in P-cluster chelation, are completely conserved as Cys12, Cys37, and Cys99 (Fig. S3). The conservation of Cys residues in IsrD and IsrK (one and three Cys residues in IsrD and IsrK, respectively) suggests that these four Cys may be involved in the holding of metal clusters such as a [4Fe-4S] cluster. It is noteworthy that in the NB protein of DPOR (BchN-BchB), one [4Fe-4S] cluster (the NB cluster) is held by three Cys in BchN and one Asp in BchB (9, 40). Thus, it cannot be excluded that a residue other than Cys in IsrD is involved in metal cluster chelation.

In addition, neither Cys275 nor His442 of the NifD subunit (*A. vinelandii*) involved in chelating FeMo-co is conserved in IsrD, suggesting that it is unlikely to hold a complex metal cluster such as FeMo-co. This is a similar feature of BchN/ChlN (DPOR) and BchY (COR) in Group V NFLs (Fig. S2).

### Reaction catalyzed by IsrBHDK

The biochemical reactions catalyzed by known NFLs implied that, in general, NFLs catalyze a variety of reductions, in which the catalytic component (homolog of NifDK) reduces diverse substrates using electrons from the reductase component (homolog of NifH) (4, 5). In addition to this implication, given that the heterologous expression of IslAB complemented the ability to use isethionate as the sulfur source in the mutant *∆DK*, it is suggested that Isr catalyzes the reductive cleavage of the C-S bond in isethionate, releasing sulfite. This cleavage reaction appears to be similar to the reaction catalyzed by methylthioalkane reductase (Mar) with respect to the reductive cleavage of the C-S bond (13). Mar converts 2-(methylthio)ethanol, giving rise to methane thiol, ethylene, and water. If it is a simple reductive cleavage of the C-S bond of isethionate by Isr, the resulting carbon product is presumed to be ethanol (Fig. 1).

### Function of IsrB

The loss of growth ability in the isethionate medium in the targeted mutant of *isrB* (*∆B*, Fig. 3) strongly indicated that IsrB is an essential protein for the reaction catalyzed by Isr. What is the function of IsrB in isethionate reduction? In the FeMo-co biosynthesis of the nitrogenase system, NifB is involved in the formation of NifB-co, the precursor of FeMo-co (3, 41). In this process, NifB catalyzes a series of complex reactions to extract a methyl group from *S*-adenosylmethionine (SAM) as the first reaction, followed by the conversion of two [4Fe-4S] clusters to NifB-co with the insertion of the methyl carbon as the central carbide of NifB-co (3, 42, 43). In the first reaction, NifB cleaves the C-S bond of SAM to release the methyl group. In this respect, it would be similar to the reductive cleavage of the isethionate C-S bond that we hypothesize that IsrHDK catalyzes. As another example, one subunit of isethionate lyase, IslB, is an RSE that acts as the activating agent for the glycyl radical enzyme IslA to form a glycyl radical on the Gly residue of IslA (28, 29). This function of the activating enzyme via radical formation is also observed in PflA, the RSE unit of pyruvate formate lyase (PflB) (44). Currently, it is unclear whether IsrB cleaves the C-S bond or activates IsrHDK. Similarly, the role of MarB in the methylthioalkane reductase system is also unclear (13). Further study from a biochemical perspective is required.

### Distribution of Isr and physiological role

**Fig 8:**
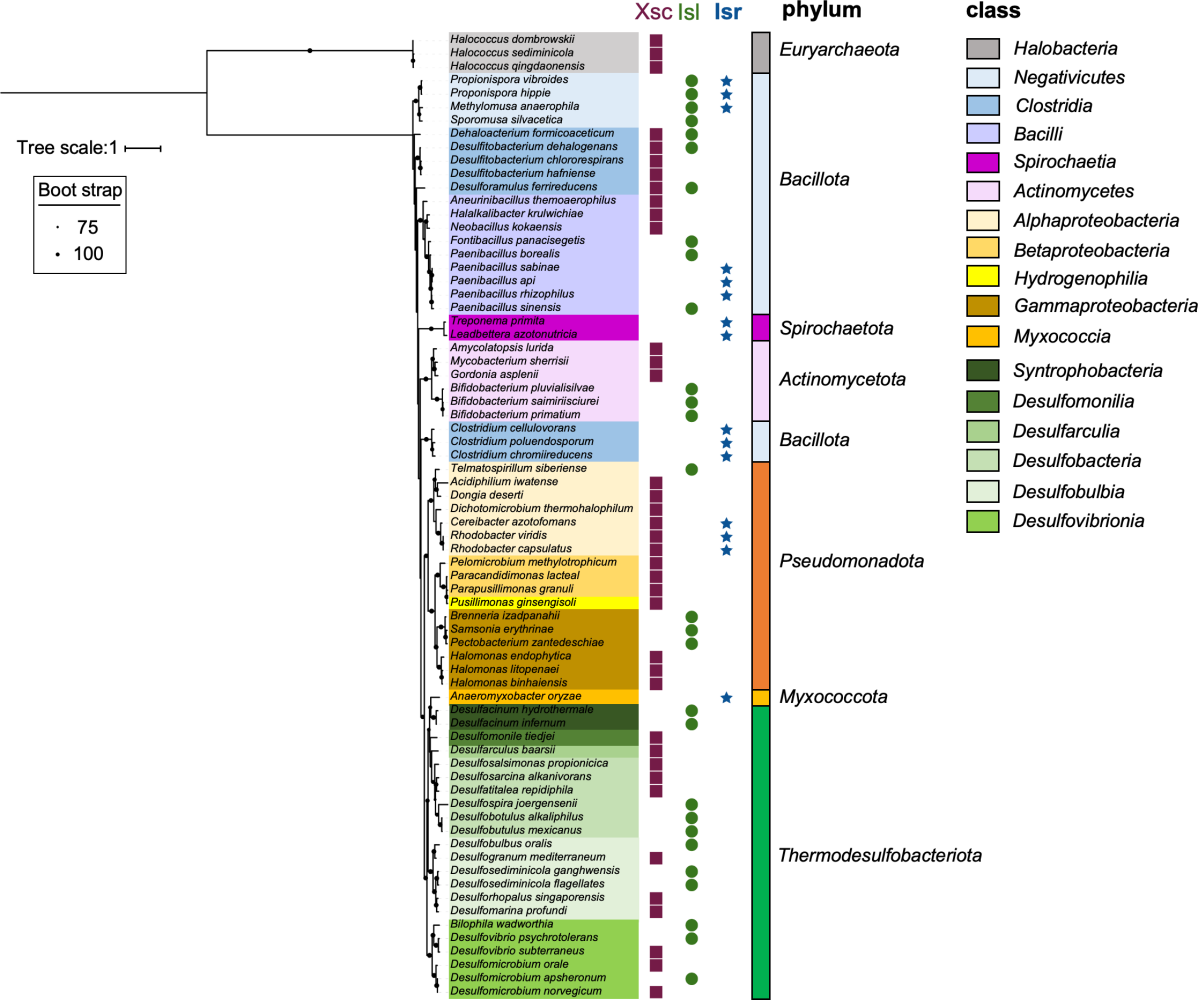
Distribution of the three oxygen-independent isethionate metabolic enzymes (Isr, Isl, and Xsc) in prokaryotes. Distribution of the three enzymes Xsc (purple squares), IslA (green circles), and IsrD (blue stars), which are oxygen-independent enzymes for isethionate metabolism, are plotted on the phylogenetic tree of 16 s rRNA. The best fit model is TIM3 +F + I + G4.

Xsc and IslAB are assimilatory enzymes that catalyze the desulfurization reactions of sulfoacetaldehyde and isethionate, respectively, in an oxygen-independent manner (Fig. 1) (27–29). In this study, we identified the genes for Isr as a third oxygen-independent desulfurizing enzyme. The distribution of these three enzymes, Xsc, Isl, and Isr, in prokaryotes shows that Isr is almost exclusively distributed in the phyla of Bacillata, Spirochaeta, Pseudomonadota, and Myxococcota, with a few exceptions (Fig. 8). We predict that these organisms carrying Isr as the sole oxygen-independent desulfurization enzyme without Xsc and IslAB can degrade isethionate to sulfite for use as a sulfur source (assimilation) or as an electron acceptor (dissimilation) under anaerobic conditions. The distribution of the three enzymes suggests that most prokaryotes carry only one enzyme for isethionate desulfurization, and organisms with multiple enzymes such as Xsc and Isr, as in *R. capsulatus*, are rare.

Based on the common biochemical reactions of nitrogenases and NFLs, Isr has ATPase activity for electron transfer from IsrH to IsrDK for isethionate reduction (3, 38). Therefore, it is suggested that the role of Isr *in vivo* is assimilation of sulfonate as a sulfur source, rather than a dissimilatory role that uses sulfonate as the electron acceptor in anaerobic respiration, as observed in IslAB of *B. wadsworthia*. Therefore, we hypothesize that Isr is used as the assimilatory enzyme by releasing sulfite from isethionate. Such a difference in physiological roles could be reflected in the difference in the distribution of those of Xsc and IslAB, which catalyze reactions that do not consume ATP.

In *E. coli*, the gene encoding oxygen-dependent isethionate degradation enzyme SsuD is transcriptionally regulated and induced only under conditions where sulfate is unavailable, suggesting that the ability to utilize taurine/isethionate as the sulfur source is advantageous for survival in environments prone to depletion of sulfate or other sulfur sources (45). The reaction catalyzed by Isr has been uncovered in this study, revealing the potential capacity of prokaryotes with only Isr and their contribution to the global sulfur cycle.

*R. capsulatus* possibly uses Xsc and Isr separately for the metabolism of taurine and isethionate, respectively. In fact, in *R. capsulatus*, the ability to metabolize isethionate was not affected in the *∆xsc* mutant, and the ability to metabolize taurine was not affected in any of the *∆isr* mutants (*∆B*, *∆H*, and *∆DK*). In addition, RNA-seq analysis showed that the Ise/Sul ratios of *xsc* and *tpa* expression levels were very low in contrast to more than 1,000 of the Ise/Sul ratios of *isrBHDK* expression (Fig. S5), indicating that isethionate addition does not significantly enhance the transcript levels of *xsc* and *tpa*. These results indicate that *xsc* and *tpa* are regulated by factors other than isethionate, even though they are located just adjacent to *isrBHDK*, which is strongly induced by isethionate. This supports the idea that the metabolic pathways for isethionate and taurine are independent in *R. capsulatus* (Fig. 7).

### Molecular phylogenetic aspects of IsrBHDK

**Fig 9:**
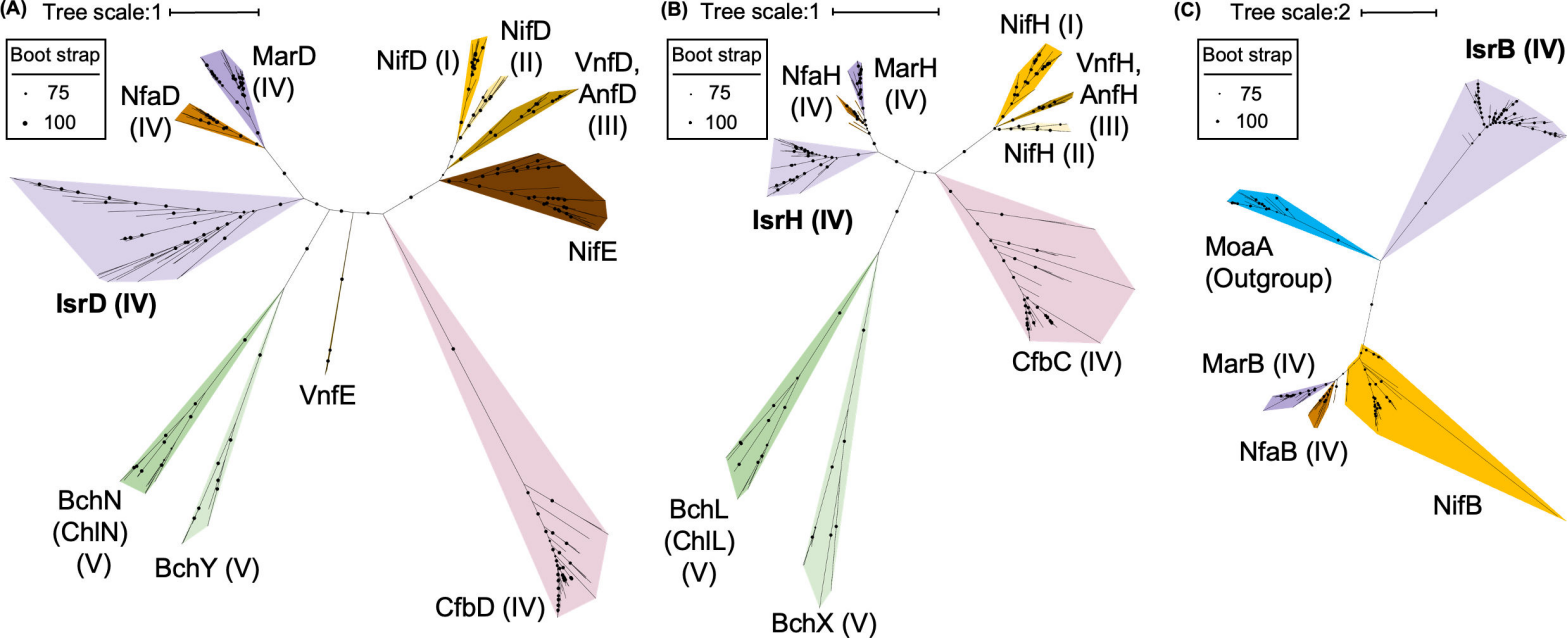
Phylogenetic trees of IsrD (**A**), IsrH (**B**), and IsrB (**C**) and their homologs. (**A**) Phylogenetic tree of IsrD and its homologs. The best fit model is Q.pfam + I + I +R8. (**B**) IsrH and its homologs. The best fit model is LG + R6. (**C**) IsrB and its homologs. The best fit model is LG + R6. Groups I–V proposed by Raymond *et al*. (4), to which each protein belongs, are shown in parentheses. NifE and VnfE were not included in the phylogenetic tree in Raymond *et al*. (4).

Molecular phylogenetic analysis of IsrBHDK was performed to understand the evolutionary relationship of this novel isethionate reductase with other NFLs and RSEs. First, a phylogenetic tree was constructed on the NifD homologs from various species. IsrD formed an independent clade in close proximity to MarD and NfaD within Group IV (Fig. 9A). A similar pattern was observed in the molecular phylogenetic trees of NifH homologs (Fig. 9B), suggesting that IsrH and IsrD originated from the common ancestral NFL sharing with MarHDK and NfaHDK within Group IV, but evolved to form independent clades. Interestingly, the phylogenetic analysis of the RSEs showed a completely different phylogenetic relationship among IsrB, MarB, NfaB, and NifB (Fig. 9C). In fact, the sequence similarity in these RSEs (IsrB, MarB, NfaB, and NifB) is limited to the radical SAM motif (Fig. S4). A blast search against the SWISS-prot database revealed that the protein with the highest similarity to IsrB was BciD, which is involved in the conversion of the C7-methyl group to the C7-formyl group in bacteriochlorophyll *e* biosynthesis (46). This phylogenetic analysis indicates that the evolutionary relationship between IsrB and NifB is different from that between IsrHDK and NifHDK. In other words, the cooperative action of NFL and RSE in Isr could be created through the recruitment of genes from independent lineages in the early evolution of life.

### Aerobic isethionate metabolism

The *isr* mutants lost the ability to grow with isethionate as a sulfur source under anaerobic photosynthetic conditions, whereas the mutants were still able to use isethionate as a sulfur source under aerobic heterotrophic conditions (Fig. S9). The isethionate-dependent growth capacity under aerobic conditions was observed even in *∆xsc*, indicating that an aerobic isethionate metabolic pathway operates independently of both the Xsc and Isr systems. SsuED is the enzyme responsible for oxygen-dependent isethionate metabolism under aerobic conditions in *E. coli*. However, no genes homologous to *ssuED* have been found in the *R. capsulatus* genome, implying that there is also a novel aerobic isethionate-metabolizing enzyme that has not been reported so far. Given that isethionate-dependent growth is completely lost under anaerobic conditions in the *isr* mutants, this aerobic enzyme (or pathway) might include oxygen-dependent reactions.

The coexistence of oxygen-independent (oxygen-labile) and oxygen-dependent (oxygen-tolerant) enzymes in isethionate metabolism is reminiscent of the coexistence of these analogous enzymes in the heme and chlorophyll biosynthesis pathways. DPOR, the NFL responsible for protochlorophyllide reduction in chlorophyll biosynthesis, coexists with an oxygen-tolerant and light-dependent enzyme; protochlorophyllide oxidoreductase (LPOR) in cyanobacteria, algae, and lower plants (47, 48). In addition, oxygen-labile anaerobic enzymes (HemN and BchE) and oxygen-dependent enzymes (HemF and AcsF) coexist in the eighth reaction (coproporphyrinogen oxidation; HemN and HemF) in heme biosynthesis (49–51) and in the reaction of protochlorophyllide formation (Mg-protoporphyrin IX monomethyl ester cyclase; BchE/ChlE and AcsF/ChlA) in the bacteriochlorophyll and chlorophyll biosynthesis systems (52–54). The coexistence of the newly identified anaerobic enzyme Isr and the presumed aerobic enzyme can be regarded as a new example of adaptive evolution to aerobic environments that emerged after the Great Oxidation Event (GOE) in the early evolution of life (55–57). Elucidation of the functions of NFLs with unknown functions is important not only for the metabolic capacity of the organisms concerned but also for elucidating ancestral metabolism and following adaptive evolution in response to the GOE.

## MATERIALS AND METHODS

### Strains and culture conditions

In this study, an adaptive strain that grows without a lag time in the RCV-isethionate medium (58) was isolated from a single colony of *R. capsulatus* SB1003 on an RCV-isethionate medium agar plate and used as the wild-type (WT). Both WT and gene-disrupted mutants were pre-cultured in 3 mL PY medium overnight and then inoculated into various media (initial OD_660_ = 0.01): RCV medium without any sulfur compounds [RCV(-S)], with MgSO_4_ (RCV-SUL), taurine (RCV-TAU), and sodium isethionate (RCV-ISE). The final concentrations of MgSO_4_, taurine, and isethionate in the RCV-SUL, RCV-TAU, and RCV-ISE media were 0.1 mM. These RCV media were prepared by substituting (NH_4_)_2_SO_4_, MgSO_4_, and FeSO_4_ with NH_4_Cl, MgCl_2_, and FeCl_3_, respectively. For anaerobic photosynthetic growth, liquid cultures were prepared in 30-mL test tubes sealed with air-tight screw caps and illuminated with a 75 W incandescent bulb (National, Osaka, Japan) at 34°C.

*R. sphaeroides* J001-1, an adapted strain for growth in Sistrom medium (59), derived from the rifampicin-resistant strain *R. sphaeroides* J001, was used in this study (60). To confirm its ability to use isethionate, *R. sphaeroides* J001-1 was cultured under anaerobic photosynthetic conditions in the PYS (61) medium for 48 hours as a preculture. Precultured cells were washed with Sistrom (-S) medium and inoculated into Sistrom (-S) medium containing 0.1 mM isethionate. Sistrom (-S) was prepared by substituting (NH_4_)_2_SO_4_, MgSO_4_, and FeSO_4_ with NH_4_Cl, MgCl_2_, and FeCl_3_, respectively.

### Construction of gene-disrupted mutants and transconjugant

Targeted gene disruption of *R. capsulatus* was performed using the homologous recombination technique, and all gene cloning operations were performed using an In-Fusion HD Cloning Kit (Takara Bio, Shiga, Japan). A conjugated pZJD29a vector (62) was employed to clone 500-bp upstream and downstream regions of the target gene. The *islAB* genes, which encode subunits of anaerobic isethionate lyase from *Bilophila wadsworthia* 3_1_6, were synthesized with codon optimization for *E. coli* (Invitrogen GeneArt, Thermo Fischer Scientific, Waltham, MA), and the nucleotide sequence of *islAB* used in this study can be downloaded from https://github.com/Yoshiki-Mo/supporting_data_2024.5. For *islA* and *islB,* an 11-bp intergenic region between *trpB* and *trpC*, present in the tryptophan synthesis operon of *E. coli,* was inserted into the intergenic region (63). The expressions of the *isrBHDK* genes (rcc02237-rcc02236-rcc02235-rcc02234) and the *islAB* genes were regulated by the *pucB* promoter that was inserted just upstream of the initial codons of *isrB* and *islA*. The chimeric gene fragments were then cloned into pBBR-MSC2 (64). For the expression of *ssuCAB* (rcc02223-rcc02224-rcc02225) or *isrBHDK* in *R. sphaeroides*, the 280-bp upstream sequence of *pucB* from *R. sphaeroides* was employed as a promoter, and the *isrBHDK* or *ssuCAB* genes were inserted downstream of the promoter unit and then cloned into pBBR-MSC2. In addition, plasmids for co-expression of *isrBHDK* and *ssuCAB* genes were constructed by tandem cloning of the respective expression gene regions just downstream of the *pucB* promoter into pBBR-MSC2. Each plasmid was transformed into cells of *R. capsulatus* and *R. sphaeroides* using the conjugative transfer method with *E. coli* S17-1 *λpir*, as described in previous studies (7). The primer sequences used in this work are listed in Table S4.

### Analysis of genes specifically induced by isethionate under photosynthetic conditions

#### RNA extraction and RNA-seq library preparation

*R. capsulatus* cells were grown in RCV-SUL or RCV-ISE medium under anaerobic photosynthetic conditions. Total RNA was extracted from cells in the exponential phase (OD_660_ = 0.2–0.3; UV-1800, Shimadzu, Kyoto, Japan). For RNA preparation, 1 mL of cells was mixed with 200 µL of Stop solution (5% phenol: 95% ethanol), vortexed for a few seconds, and then centrifuged quickly (13,300 rpm, AR015-24, TOMY, Tokyo, Japan). Cells were resuspended in TE buffer (100 µL) containing 10 mg/mL lysozyme and incubated at room temperature for 5 minutes. RNA was extracted using NucleoSpin RNA (Macherey-Nagel, Düren, Germany) according to the manufacturer’s protocol. Genomic DNA was on-column digested with RNase-free DNase treatment. The concentration of total RNA was determined by measuring the absorbance at 260/280 nm using a NanoDrop spectrophotometer (Thermo Fischer Scientific). Library preparation and stranded RNA-seq were performed by Genome-Lead (Takamatsu, Japan). In brief, the MGIEasy RNA Directional Library Prep set (MGI) was used for reverse transcription and subsequent cDNA library preparation. Sequencing was performed using the DNBSEQ-G400RS sequencer, and the 26–41 million sequencing reads obtained from each replication were used for subsequent RNA seq data analysis (Table S5).

#### RNA-seq

Demultiplexed raw sequencing reads were quality-trimmed and adapter-filtered using fastp (65) with default settings. Quality-filtered reads were aligned to the *R. capsulatus* SB1003 genome (GenBank accession: GCA_000021865.1) using Bowtie2 (66) with global alignment and sensitive settings. SAM alignments were converted to the binary sam (BAM) format using the SAMtools sort command (67). The number of sequencing reads aligned to each open-reading frame was counted using FeatureCounts with the option “-p -T 8 t CDS -g gene_id” (Table S6) (68). Differential expression gene (DEG) analysis between RCV-SUL and RCV-ISE conditions was performed using the Bioconductor DESeq2 v.1.3.4 package (Figs. S10 and S11; Tables S7 and S8) (69). The *P*-values were adjusted for multiple testing using the Benjamini–Hochberg method to control the false discovery rate (FDR) below 0.001. Genes significantly induced in RCV-ISE conditions with FDR < 0.001 and fold change greater than 100-fold compared with RCV-SUL conditions are shown in Table 1; Fig. S5. Volcano plots were generated using the ggVolcanoR shiny server (Fig. S11) (70).

### Biochemical analysis of IsrD and IsrK

#### Plasmid construction for expression of 6xHis-IsrD and IsrK

IsrD was overexpressed as a fusion protein with a 6xHis-tag at the N-terminus. The gene for 6xHis-IsrD was inserted into the native operon, *isrB-isrH-isrD-isrK*, instead of *isrD*, and the chimeric operon controlled by the *pucB* promoter was constructed on the shuttle vector pBBR-MSC2. This plasmid was then transformed into *R. capsulatus* Δ*isrDK* cells. The primer sequences used in this study are listed in Table S4.

#### Preparation of crude extracts

The transconjugant for the expression of the 6xHis*-isrD* gene was pre-cultured in 3 mL PY medium overnight under aerobic condition and then inoculated into a 1 L RCV-ISE medium. The transconjugant was grown for 4 days in a screw-capped 1-L bottle under the anaerobic photosynthetic condition. The cells were then harvested by centrifugation at 8,000 rpm for 10 minutes at 4°C (RPR10-2, Himac, Japan). All subsequent procedures were carried out in an anaerobic chamber (model A, COY, Grass Lake, MI) using solutions that had been degassed and stored in an anaerobic chamber, with 1.7 mM sodium dithionite (final concentration) added just before use to remove residual oxygen. Cell pellets were suspended in 5 mL lysis buffer (8) and the cells disrupted by four 30-s sonication bursts at 50% output (Sonifier 250 sonicator with a micro tip; Branson, Danbury, CT). The sonicate was then centrifuged at 45,000 rpm (S50A, Himac, Japan) for 1 hour at 4°C. The resultant supernatant fractions were collected as crude extracts and stored at 4°C for protein purification.

#### Mass spectroscopy of the 6xHis-IsrD and IsrK proteins

The purification procedures for 6xHis-IsrD were carried out in an anaerobic chamber using solutions that had been degassed and stored in an anaerobic chamber, with 1.7 mM sodium dithionite (final concentration) added just before use to remove residual oxygen. Aliquots of 10 mL crude extracts were loaded onto an Ni-NTA column (1 mL, GE Healthcare) that was equilibrated with the 5 mL binding buffer (20 mM Tris-HCl; pH 7.4, 500 mM NaCl, 5 mM imidazole). After the column was washed with 10 mL of binding buffer, the 6xHis-tagged protein was eluted by 2 mL elution buffer with varying concentrations of imidazole (50 mM, 100 mM, 250 mM, or 500 mM imidazole, in 20 mM Tris-HCl; pH 7.4 and 500 mM NaCl), respectively. The fractions eluted with the elution buffer containing 250 mM imidazole were collected as the purified fraction. Protein concentrations were determined using the Protein Assay Rapid Kit Wako II (FUJIFILM, Osaka) with bovine serum albumin as a standard. The purified proteins were separated by SDS-PAGE, and each band of the purified protein was excised and subjected to mass spectrometry analysis, as described in previous studies (71).

#### Absorption spectra of the 6xHis-IsrD-IsrK protein

To prepare the co-purified fraction of 6xHis-IsrD and IsrK, crude extract preparation and protein purification were conducted according to the methods in the previous sections. However, dithionite was not added during the purification in order to prepare the co-purified fraction of 6xHis-IsrD and IsrK as an as-isolated fraction (without dithionite). The sample was placed in a cuvette that was tightly screw-capped, and absorbance spectra were recorded (V-660, Shimadzu, Kyoto). For preparing the reduced form of the IsrDK protein, dithionite was added to the sample at a final concentration of 5 mM in the anaerobic chamber, and then the absorbance was recorded as the reduced fraction.

### Phylogenetic analysis of NFLs and RSEs

#### Search for a single ortholog cluster of Group IV

The determination of orthologs of the various proteins in Group IV NFL was performed using the following two-step procedure. Since the Group IV NFLs Isr, Mar, and Nfa comprise the BHDK homolog set and Cfb comprises the HD homolog set, respectively, strains carrying all of the genes in these sets were defined as strains harboring the respective NFLs (Table S9).

##### Search for homologs

Regarding Isr, homologs of IsrB (WP_013067946.1), IsrD (WP_013067944.1), and IsrK (WP_013067943.1) were BLASTp searched against the NCBI RefSeq Select proteins database with an e-value cutoff of 1e^−10^ and a minimum alignment fraction of 70%. Regarding IsrH (WP_013067945.1), IsrH was retrieved from strains carrying all IsrB, IsrD, and IsrK orthologs. For homologs of other Group IV NFL (Cfb, Nfa, and Mar) subunits, the orthologs of the respective D subunits were first defined, and the homologs of other subunits (B, H, and K in Mar and Nfa, and H in Cfb) were searched for species carrying the respective D subunit ortholog.

##### Phylogenetic inference of the diverse Group IV NFLs and related sequences

To estimate the phylogenetic relationship of Group IV NFL subunits and evolutionarily related protein families, phylogenetic analysis was performed as follows. First, each homolog sequence set was multiple sequence-aligned using Mafft version 7.0 with default settings (72). Multiple sequence alignments were visually checked, and highly gapped positions were trimmed using Clipkit version 1.4.1 with default settings (73). Maximum likelihood (ML) phylogenetic inference was performed with 1,000 ultra-fast bootstrap replicates, and Modelfinder was used to estimate the best-fit substitution model (74) using IQ-TREE version 2.2.0.3 (75). The resulting tree was visualized using iTOL v.6.0 (Fig. S12) (76). The monophyletic clade containing known Group IV NFL and related sequences was manually recovered and treated as a single ortholog cluster.

### Phylogenetic inference of the NFL

Phylogenetic analysis of each single ortholog cluster (subunits of NFLs) was iteratively performed on the following sequences; 1) Groups I, II, III, and V NFL orthologs, as described in (4) and (77), and 2) Group IV NFL orthologs explored in this study (Table S9). ModA, which was used as an outgroup in the estimation of the B subunit phylogeny, was obtained from the NCBI UniProtKB/SWISS-Prot database (Table. S10). For ML phylogenetic inference, IQ-TREE was used as explained in the previous section. The treeline used to create the phylogenetic tree can be downloaded from https://github.com/Yoshiki-Mo/supporting_data_2024.5.

### Distribution of isethionate-metabolizing enzymes

#### Search for a single ortholog cluster of isethionate-metabolizing enzymes

The homologs of the isethionate-metabolizing enzymes Xsc and IslA were BLASTp searched against the NCBI RefSeq Select protein database with an e-value cutoff of 1e^−10^ and a minimum alignment fraction of 70%. The top 1,500 hits with zero or the lowest e-value were retrieved. Following the method described earlier, sequences from the query-containing monophyletic clade were considered as single ortholog clusters and manually recovered (Table S11).

#### Phylogenetic inference of strains carrying isethionate-metabolizing enzymes

The phylogenetic relationship among strains containing isethionate-metabolizing enzymes was inferred using SSU 16S rRNA sequences. Full-length 16S rRNA sequences were obtained using Barnap version 0.9 (https://github.com/tseemann/barrnap) from each genome assembly or manually extracted from the LPSN website (October 17 in 2023 access) (78). A 1,000 bootstrap replication ML tree estimation was performed according to method given in the previous section. The treeline used to plot the phylogenetic tree is available at https://github.com/Yoshiki-Mo/supporting_data_2024.5.

### Comparative genome analysis for genome profiling

The genome sequences of 14 *R. capsulatus* and 29 *R. sphaeroides* strains were downloaded from the NCBI assembly (December 13, 2022 access). Genomes satisfying completeness >90% and contamination <5% for 120 conserved proteins according to the MIMAG (meta)genomic criteria were selected (79). The remaining 41 high-quality (HQ) genomes were confirmed for genome taxonomy using two methods. First, the ANI distances of the 41 genomes against *R. capsulatus* SB1003 (GCA_000021865.1) and *R. sphaeroides* type strains (GCA_000012905.2) were checked using Pyani version 0.2.11 (80). Second, the phylogenetic placement of 41 HQ genomes was investigated using the genome taxonomy tool kit GTDB-tk version 2.3.0 (81, 82). Using these two methods, eight strains were classified as *R. capsulatus*_B, five as *R. capsulatus*, and 17 as *Cereibacter_*A *sphaeroides*. The remaining 30 genome sequences (Fig. S7; Tables S1 and S2) were annotated using prokka with default settings (https://github.com/tseemann/prokka). The resulting GFF files were used to search for core genes specifically conserved in all of the eight *R. capsulatus_*B strains that retained the Isr genes but not conserved in the 17 *C_*A*. sphaeroides* strains and the five *R. capsulatus* strains that did not retain the Isr gene.

Pan-genome analysis was performed using PIRATES with the “-s 60, 70, 80, 90, 95 a” option (83). The Scoary package was employed to identify shared genes within a species of *R. capsulatus*_B containing Isr, which is assumed to possess metabolic capability for isethionate (84). Statistical significance was determined at *P* < 0.01 after applying Bonferroni multiple test correction (Table S12).

## Data Availability

Raw RNA-seq sequencing reads have been deposited in the DDBJ Sequence Read Archive (DRA) under BioSample accession numbers SAMD00770669. Data are contained within the article or Supplementary Material. All raw data have been deposited into the data managing system in Nagoya University.

## SUPPLEMENTAL MATERIAL

Supplemental figures msphere.00498-24-s0001.pdf Figures S1-S12.

Supplemental tables msphere.00498-24-s0002.xlsx Tables S1-S12.

ASM does not own the copyrights to Supplemental Material that may be linked to, or accessed through, an article. The authors have granted ASM a non-exclusive, world-wide license to publish the Supplemental Material files. Please contact the corresponding author directly for reuse.
